# Supplementary material for: Engineering an Optimal Y280-Lineage H9N2 Vaccine Strain by Tuning PB2 Activity
Source: Int J Mol Sci. 2023 May 16;24(10):8840. doi: 10.3390/ijms24108840 (PMC10218604; doi:10.3390/ijms24108840)
Supplement: Supplementary file 1 [file ijms-24-08840-s001.zip › ijms-2265695-supplementary.pdf]

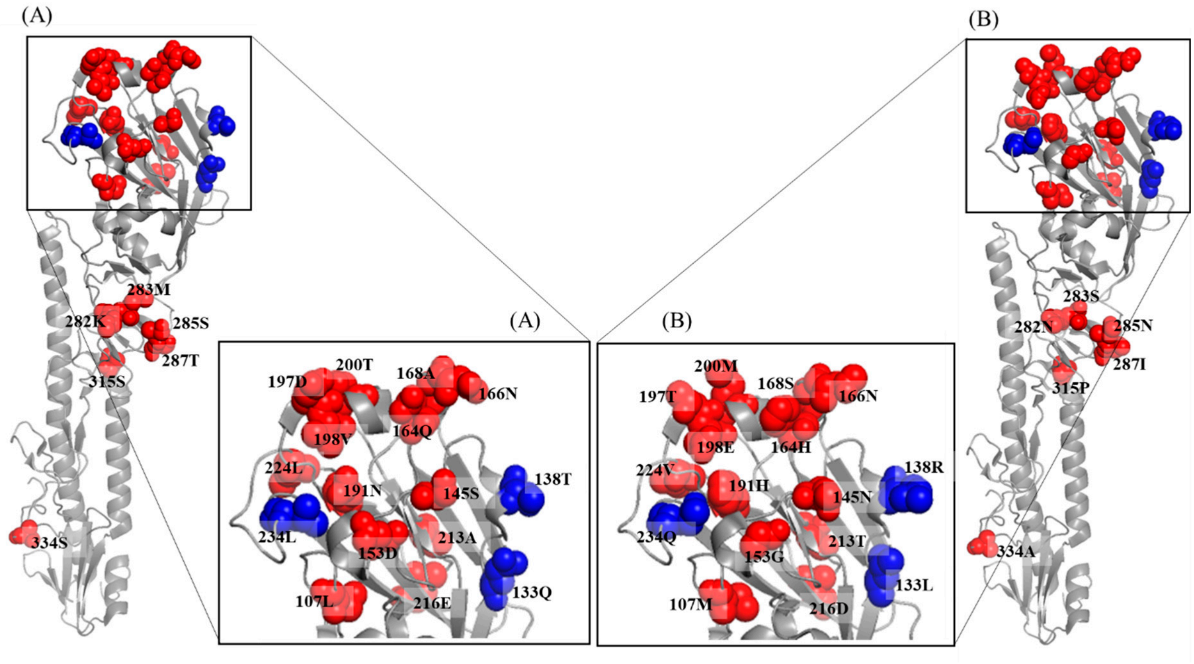

Figure S1. Localization of different amino acid residues composing epitopes of hemagglutinin. The 1jsd PDB file was used to generate 3D structures of (A) SL20 and (B) 01310 CE20 using PyMOL. The amino acid residues composing epitopes that differ between SL20 and 01310 are colored in red (referenced in 1) and blue (referenced in 2).

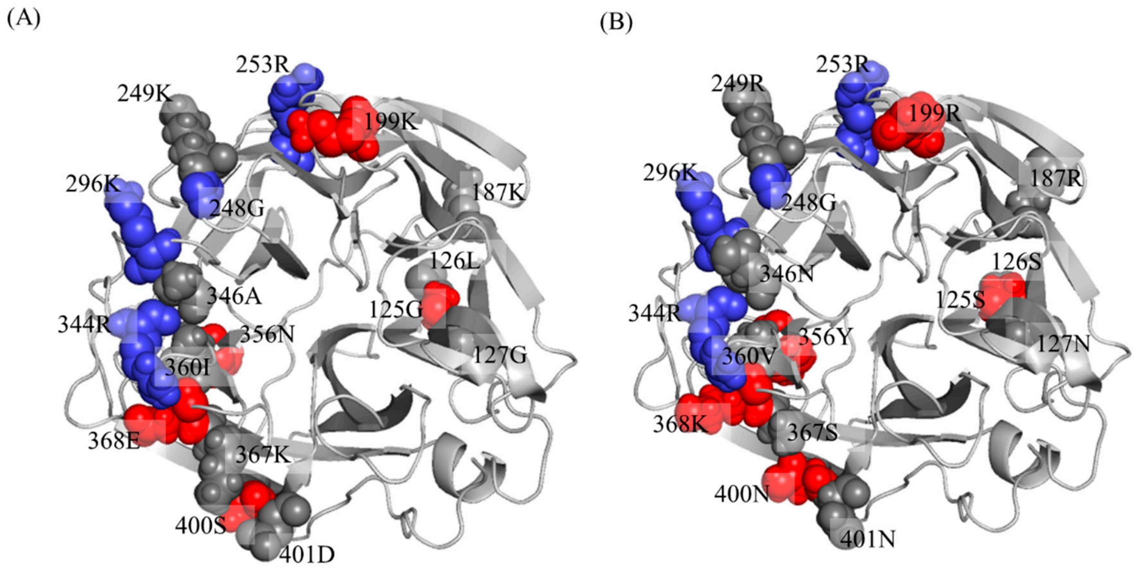

Figure S2. Localization of different amino acid residues composing epitopes of neuraminidase. The top view 3D structures of (A) SL20 and (B) 01310CE20 were generated using PyMOL based on the inn2 PDB file. The amino acid residues composing epitopes that are same or different between SL20 and 01310CE20 are colored in blue and red, respectively. Adjacent amino acid residues that differ between the two strains are shown in black.

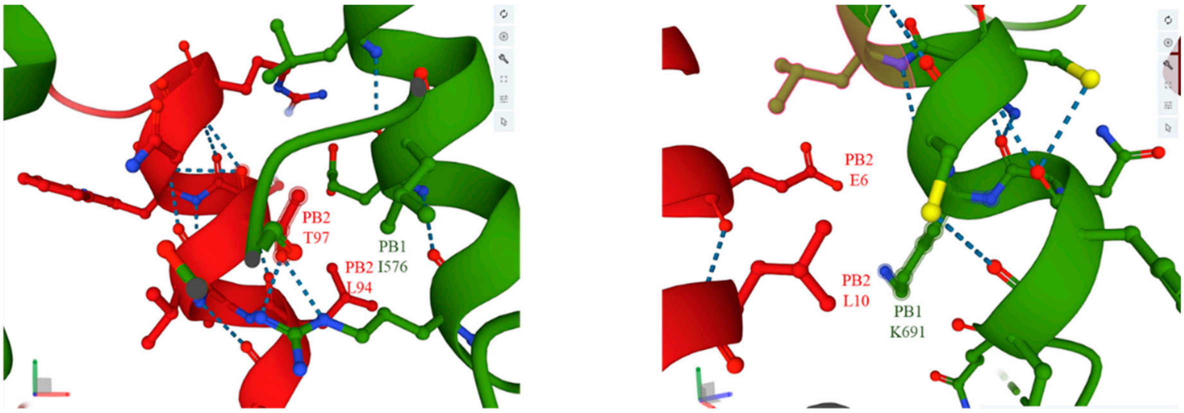

PR8/01310: PB2 L94

PR8/01310: PB2 E6, L10

Figure S3. Identification of variable PB1 amino acid residues interacting with L94 (left) and L10 (right) residues of PB2.

Table S1. Nucleotide and amino acid identities of most similar Korean and Chinese H9N2 strains with SL20wt.

Gene	A/Korean_native_chicken/South Korea/N20-99/2020			A/chicken/Shandong/1844/2019		
	Nucleotide identity (%)	Amino acid identity (%)	Accession no.	Nucleotide identity (%)	Amino acid identity (%)	Accession no.
PB2	99.52	99.74	MT944186.1	98.73	99.21	MN765144.1
PB1	99.47	99.74	MT944187.1	99.12	99.08	MN765145.1
PB1-F2	99.63	98.90	MT944187.1	98.90	96.70	MN765145.1
PA	99.67	99.58	MT944188.1	99.07	98.88	MN765146.1
PA-X	99.74	100.00	MT944188.1	99.34	99.60	MN765146.1
HA	99.29	98.75	MT944189.1	98.75	98.39	MN765147.1
NP	99.27	99.60	MT944190.1	98.73	99.00	MN765148.1
NA	99.36	99.36	MT944191.1	98.72	98.93	MN765149.1
M1	99.79	100.00	MT944184.1	99.58	99.68	MN765150.1
M2	99.87	100.00	MT944184.1	99.74	100.00	MN765150.1
NS1	99.71	99.57	MT944193.1	98.99	98.70	MN765151.1
NEP	99.75	99.62	MT944193.1	98.99	98.49	MN765151.1

Table S2. Mutations increasing mammalian pathogenicity of SL20wt.

Genes	Mutation of SL20wt	References
PB2	I66M	[15]
	I109V	[15]
	I133V	[15]
	**I292V**[c]	[40]
	A588I/T/V	[45]
	**V598T/I**[c]	[42]
PB1	368V	[41]
PA	356R	[46]
	409N	[39]
	T129I	[24]
	G351E	[24]
	M628V	[24]
HA[a]	RSSR	-
	155T	[44]
	183N	[44]
	190V	[44]
	**Q226L**[c]	[44]
NA[b]	**Stalk deletion (63-65 aa)** [c]	-
M1	**N30D**[c]	[38]
	**T215A**[c]	[38]
NS1	**P42S**[c]	[43]
	G139D/N	[25,26]
	S151T	[25,26]
	**Nonsense mutation at 218th codon (truncation of NA from 237 to 217 aa)** [c]	

[a] H3 numbering [A/Aichi/2/1968(H3N2)]

[b] N2 numbering [A/Aichi/2/1968(H3N2)]

[c] Common MPMs found in most genotype S H9N2 viruses were represented in bold.

Table S3. Accumulation of mutations in the second sialic acid binding site of NA subtype 2.

**Virus**	**2nd-sialic acid binding site of N2 protein**		
	**370-loop**	**400-loop**	**430-loop**
A/turkey/Minnesota/78	ISKDSRSG	DNNNWS	RPQE
01310 CE20	--------	------	----
F/98	-K------	-SD---	----
Egypt/F10533D	-K----A-	-SDG--	----
SL20	-KNG----	-SDD--	----
SD1844	-KNG----	-SDD--	----

Table S4. Primer sets used in this study

Primer	Sequece (5’ – 3’)
cmv-SF	5′-TAAGCAGAGCTCTCTGGCTA-3′
bGH-SR	5′-TGGTGGCGTTTTTGGGGACA-3′
SL20-H9-L226Q-F	5′-CCTCTTGTCAACGGTCAGATGGGAAGAATTG-3′
SL20-H9-L226Q-R	5′-CAATTCTTCCCATCTGACCGTTGACAAGAGG-3′
